# Overuse and Underuse of Antiosteoporotic Treatments According to Highly Influential Osteoporosis Guidelines: A Population-Based Cross-Sectional Study in Spain

**DOI:** 10.1371/journal.pone.0135475

**Published:** 2015-08-28

**Authors:** Gabriel Sanfélix-Gimeno, Isabel Hurtado, José Sanfélix-Genovés, Cristóbal Baixauli-Pérez, Clara L. Rodríguez-Bernal, Salvador Peiró

**Affiliations:** 1 Health Services Research Unit, Center for Public Health Research (FISABIO), Valencia, Spain; 2 Red de Investigación en Servicios de Salud en Enfermedades Crónicas (REDISSEC), Valencia, Spain; 3 Fundación de Investigación del Hospital Clínico Universitario—Instituto de Investigación Sanitaria INCLIVA. Valencia, Spain; Van Andel Institute, UNITED STATES

## Abstract

Inappropriate prescribing of antiosteoporotic medications has been observed; however, the joint study of both overuse and underuse has barely been attempted. Spain, with its high utilization rates, constitutes a good example to assess differences in over and under use according to diverse highly-influential osteoporosis guidelines (HIOG) worldwide. We used data of a population-based cross-sectional study including 824 post-menopausal women ≥50 years old living in the city of Valencia, Spain and aimed to estimate the percentage of women eligible for treatment, and the proportion of overuse and underuse of antiosteoporotic treatment according to HIOG. The prevalence of antiosteoporotic treatment in postmenopausal women ≥ 50 in Valencia was 20.9% (95%CI:17.6–24.4). The type of antiosteoporotic drugs prescribed varied greatly depending on the medical specialty responsible of the initial prescription. When applying the HIOG, the percentage of women 50 and over who should be treated varied from less than 9% to over 44%. In real terms, from the approximately eight million women of 50 years old and over in Spain, the number eligible for treatment would range from 0.7 to 3.8 million, depending on the guideline used. A huge proportion of inappropriate treatments was found when applying these guidelines to the Spanish population, combining a high overuse (42–78% depending on the guideline used) and underuse (7–41%). In conclusion, we found that the pharmacological management of osteoporosis in women of 50 and over in this population combines an important overuse and, to a lesser extent, underuse, although the level of inappropriateness varied strikingly depending on the CPG used. It seems urgent to reduce treatment overuse without neglecting underuse, as is urgent an attempt to reach wider agreement worldwide regarding osteoporosis management, in order to facilitate appropriate treatment and development of policies to reduce effectively treatment inappropriateness.

## Introduction

The most widespread definition of underuse is *“the failure to provide a health care service when it would have produced a favourable outcome for a patient”* [1], while overuse occurs “*when a health care service is provided under circumstances in which its potential for harm exceeds the possible benefit*” [1], although recently it has been pointed out that overuse could include different dimensions in relation to risk-benefit, cost-benefit and patient preference approaches [2]. While research has traditionally focused on identifying and reducing the underuse of appropriate services in patients with a specific condition (e.g. the use of antiplatelet agents in secondary prevention of ischemic heart disease), the overuse of health care services has become an increasingly recognized but understudied problem [3,4]. Nonetheless, recent reviews have shown high rates of overuse for a range of diagnostic tests, imaging tests and therapeutic services in the US setting [4–7].

Overuse and underuse require operational definitions for each set of the patient’s clinical condition and the service provided (**Fig A in S1 File**). Criteria for these definitions may come from clinical trials, but more frequently come from expert consensus [8] or from the criteria established in clinical practice guidelines (CPG) [9]. However, the latter may exhibit some variability in their recommendations [9,10]. In any case, and regardless of the method used, the assessment of overuse and underuse requires having sufficient information to apply the appropriateness criteria to each of the patients evaluated. This is particularly important in the identification of underuse because it requires population samples of non-treated patients with sufficient information to assess treatment appropriateness, and in many cases these patients have no specific information or may even have no contact at all with the healthcare system. Therefore, availability of information allowing underuse assessment is extremely valuable.

Furthermore, studies have usually shown the overuse of specific services in a particular condition or the underuse of other services in a different condition (**Fig A in S1 File**). However, overuse and underuse may concur in the same healthcare service and in the same clinical condition.

Regarding data on use of antiosteoporotic treatment, while Spain is one of the European (and worldwide) countries with a lower incidence of osteoporotic fracture [11,12], antiosteoporotic medications are widely prescribed. A recent report analyzing the variability in the consumption of several therapeutic drugs in 15 developed countries (including the U.S., Canada, and several European countries) identified Spain as the country with the highest utilization rates of antiosteoporotic drugs [13]. In addition, temporal trends show a very rapid and disproportionate growth in osteoporosis drug consumption in recent years [14]. Concerning the appropriateness of antiosteoporotic drugs, previous studies suggest that Spain [15,16] and other countries [17] are witnessing a massive use of these treatments in young women with a very low risk of fracture, while there is a significant underuse in women (and men) at a high risk of fracture, including those who have already suffered a major osteoporotic fracture. These estimations, however, could vary according to the criteria used to assess over and/or underuse. Given the wide range of clinical practice guidelines on osteoporosis existing globally, it would be desirable to determine the extent to which estimations change according to such guidelines, and a setting with high utilization rates of antiosteoporotic treatment could serve as a good example.

The FRAVO study is a population-based cross-sectional study designed to estimate the prevalence of vertebral fracture and densitometric osteoporosis among post-menopausal women over 50 years old living in Valencia (Spain)[18,19]. The comprehensive information collected allows the estimation of the risk of fracture and the operationalization of the criteria for antiosteoporotic prescribing used in most CPGs, enabling the assessment of the impact on the population of using different guidelines, as well as estimating the population over or underuse of these treatments according to the criteria of each CPG. In this study, we aimed to: 1) describe the population prevalence of antiosteoporotic treatment among post-menopausal women of 50 and over and the possible associations with socioeconomic factors, individual fracture risk factors and the 10-year risk of hip fracture (assessed by FRAX [20]), 2) estimate the impact on the population of using different international and national guidelines regarding antiosteoporotic treatments and, 3) estimate the over and underuse of antiosteoporotic treatments among post-menopausal women of 50 and over according to the criteria established by these guidelines.

## Methods

### Design

Population-based cross-sectional study conducted between February 2006 and March 2007, primarily designed to estimate the population prevalence of vertebral fracture and densitometric osteoporosis among post-menopausal women of 50 and over in the city of Valencia, Spain [18,19].

### Population and Sample

The study population was post-menopausal women of 50 years old and over living in the city of Valencia, Spain, excluding women with cognitive impairment, physical impediment preventing a woman from going to the radiology center by her own means, race other than Caucasian and unwillingness to participate in the study. The methods and main results of the FRAVO study have been fully described elsewhere[18,19,21]. Briefly, from an age-stratified random sample of 1758 women resident in Valencia, a total of 824 fulfilling inclusion and exclusion were included. Twenty cases for whom the X-Ray, the densitometry or the BMI was not available were excluded in some analyses. As the final sample did not exactly fit the population age distribution of the women of 50 and over in Valencia, some estimates were weighted according to that population age distribution in 2006.

### Variables and definitions

Information about socio-demographic characteristics, lifestyle and risk factors for vertebral fracture collected using the interviewer-administered questionnaire included, among other variables, the subject's age, educational level, body mass index, early menopause (defined as menopause before the age of 40), history of parental hip fracture, prior non-vertebral osteoporotic fracture, treatment with glucocorticoids (use of oral glucocorticoid for at least 3 months in the previous year) or other drugs that decrease bone mass (at least one prescription of lithium, anticonvulsants, high dose thyroxin or immunosuppressive treatment in the previous year), smoking, dietary calcium intake, and secondary causes of osteoporosis (gastrectomy, bowel resection, inflammatory bowel disease, thyroidectomy, diabetes mellitus, chronic liver disease, chronic obstructive pulmonary disease, rheumatoid arthritis, transplantation, chronic kidney failure). Spinal radiographs were performed using standardized techniques and two radiologists, who were blind to all data concerning the patients, performing the semi-quantitative evaluation of the radiographs using the Genant method [22,23]. Densitometric examinations were performed with two calibrated densitometers and the World Health Organization (WHO) osteoporosis classification criteria based on T-scores [24] were used to classify bone mineral density (BMD) results as normal, osteopenia or osteoporosis. Using the FRAX tool calibrated for Spain (www.shef.ac.uk/FRAX/index.htm) the 10-year risk of hip and major fracture was estimated for each patient [20]. Regarding antiosteoporotic medication, information was recorded on current treatments (bisphosphonates, raloxifene, strontium ranelate, teriparatide, hormone replacement therapy and calcitonins, which were the antiosteoporotic agents available in Spain during 2006–7), duration of treatment and the specialty (general practitioner, orthopaedic surgeon, gynaecologist, rheumatologist, and other/unknown) of the prescriber of the first antiosteoporotic treatment.

### Selection of guidelines and operational criteria

We revised the guidelines chosen for inclusion in a previous review [25] and selected the closest to 2007 versions of four international guidelines (National Institute for Health and Care Excellence (NICE, UK)[26,27], National Osteoporosis Foundation, (NOF, US)[28]; National Osteoporosis Guideline Group (NOGG, UK) [29]; and Osteoporosis Canada [30]), and six Spanish guidelines (Spanish Society for Family and Community Medicine (semFYC)[31], Spanish National Health System (SNS)[32], Spanish Society for Bone Research and Mineral Metabolism (SEIOMM) [33], Spanish General Medical Society (SEMERGEN) [34], Spanish Orthopaedic Surgery and Traumatology Society (SECOT) [35], and the Spanish Rheumatology Society (SER) [36]). These CPGs are, from the authors’ point of view, the most well known and influential in the Spanish setting, and many of them are also highly influential globally. This selection was also based on a survey to around 75 professionals of different specialities who rated the different guidelines according to their influence in their clinical practice. This choice did not take into account the quality of CPGs development and does not involve any judgment about the quality or validity of these CPGs compared to other guidelines, nor any endorsement from the authors. The guidelines criteria are described in **Table A in S1 File**. Some vague criteria were unambiguously defined to allow their use in the study databases (the corresponding specifications are also included in the **Table A in S1 File**).

### Ethical Aspects

The study was approved by the Institutional Review Board of the Primary Care Departments of Valencia and Castellon. All of the participating women were informed of the study’s characteristics and risks, and all gave signed informed consent prior to enrolment.

### Analysis

First, we briefly described the clinical and demographic characteristics and treatment rates of the participating women and conducted bivariate analyses to determine which characteristics were related to osteoporosis treatment. We also described the drugs used and the medical speciality of the physician who prescribed the first antiosteoporotic treatment. Second, we used a multivariable logistic regression (backward-forward stepwise method, with p<0.05 for entrance and p<0.10 for removing variables) to retain the variables independently associated with receiving osteoporosis treatment. Third, we used the information from participants in the FRAVO study to estimate the percentage of women aged 50 years and over who would be recommended for treatment according to the respective guidelines (impact on the population), with the corresponding 95% confidence intervals (95% CI), calculated using the binomial approach.

Finally, we assessed the inappropriateness of treatments: the proportion of treated women without a treatment recommendation according to the respective CPGs (overuse) and the proportion of non-treated women with a positive recommendation for treatment according to the above-mentioned guidelines (underuse). All the analyses were performed using the STATA 11.0 (Stata Corp) statistical software.

## Results

**Fig 1:**
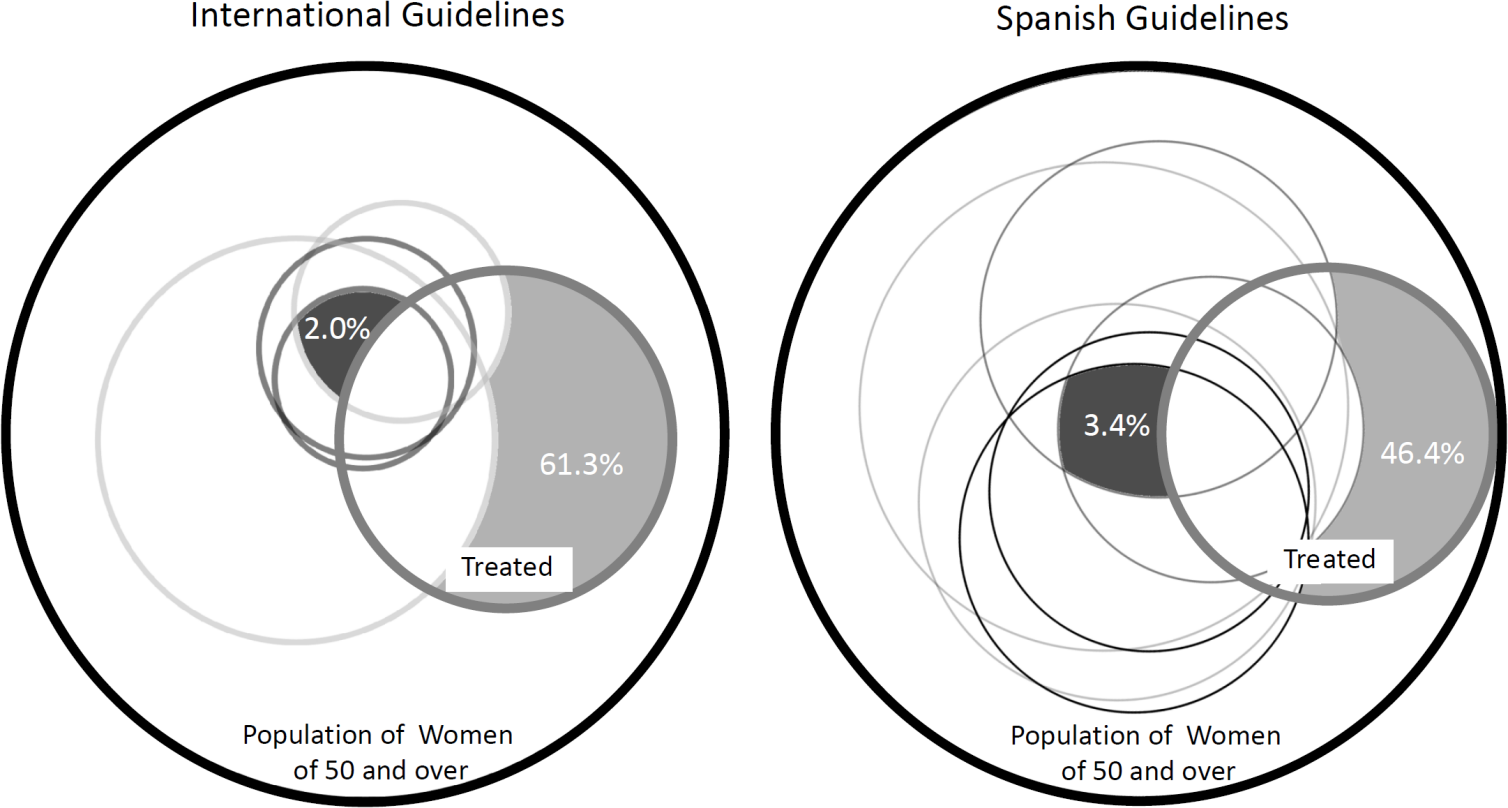
Overuse and underuse of osteoporotic treatment in women of 50 and over. The black external circle indicates the total population of women aged 50 and over and the thick gray line circle the proportion of women treated. Each one of other circles represents women who should be treated according to different international (left) or Spanish (right) guidelines. The light gray area denotes the percentage of women treated who do not require treatment (overuse) according to either all international or Spanish guidelines. The dark gray area denotes the percentage of untreated women requiring treatment according to either all international or Spanish guidelines.

The study population included 824 post-menopausal women of 50 years old and over living in the city of Valencia, Spain. Of these, 186 (22.0%) were on antiosteoporotic treatment. After weighting the sample according to the population age structure of Valencia, the estimated prevalence of antiosteoporotic treatment in postmenopausal women of 50 years old and over in Valencia was 20.9% (95%CI: 17.6–24.4). The most commonly prescribed drugs were alendronate (36.6%), risedronate (24.7%) and raloxifene (22.5%), followed by HRT (9.1%), calcitonins (3.7%), strontium ranelate (2.2%) and PTH (1.1%). Regarding the origin of the initial prescription, orthopaedic surgeons were responsible for most of them (37.1%), followed by gynaecologists (32%), general practitioners (19.4%) and rheumatologists (8.1%). Fig 1 shows the antiosteoporotic drugs prescribed according to the medical specialty responsible for the initial prescription. Orthopaedic surgeons prescribed risedronate as their first choice (40.6% of their treatments), followed by alendronate (36.3%), but prescribed calcitonins and PTH more frequently than other specialties; gynaecologists prescribed primarily raloxifene (40.0%), followed by hormone replacement therapy (26.7%) and alendronate (21.7%); general practitioners preferentially prescribed alendronate (58.3%), followed by risedronate (22.2%) and raloxifene (16.7%); and for rheumatologists, their first choice was alendronate (40.0%), followed by risedronate (26.7%), raloxifene (20.0%), and strontium ranelate (13.3%), the latter being prescribed mainly by this speciality.

Regarding the sociodemographic, lifestyle, and clinical characteristics considered (Table 1), age at menopause, bone mineral density (BMD), morphometric vertebral fracture, BMI and fracture risk score, were associated to antiosteoporotic prescribing. Women with early menopause, densitometric osteoporosis and moderate or severe morphometric vertebral fractures were more likely to be treated (33.8%, 30.2% and 42.0%, respectively). Regarding the 10-year risk of hip fracture (assessed by FRAX), the proportion of women treated was higher for those with moderate (1–3) risk scores (28.8%). Obese women had lower treatment rates (15.0%).

**Table 1:** Characteristics and antiosteoporotic use of the study population^a^.

		n (%)	Treated (%)	p^b^
Age	50–54 years	111 (13.5)	16.2	0.27
	55–59 years	156 (18.9)	21.2	
	60–64 years	173 (21.0)	23.7	
	65–69 years	170 (20.6)	27.7	
	70–74 years	144 (17.5)	19.4	
	75+ years	70 (8.5)	20.0	
Educational level	No studies	155 (22.1)	20.0	0.81
	Primary	350 (49.9)	22.6	
	Secondary/Univers.	196 (28.0)	21.9	
BMI	<20	14 (1.7)	35.7	0.006
	20.0–24.9	173 (21.0)	27.8	
	25.0–29.9	349 (42.4)	24.4	
	≥30	287 (34.9)	15.0	
Menopause age ≤40 y	No	754 (91.6)	20.2	<0.001
	Yes	69 (8.4)	42.0	
BMD	Normal	168 (20.4)	16.1	0.001
	Osteopenia	423 (51.4)	19.9	
	Osteoporosis	232 (28.2)	30.2	
Parental history of osteoporotic fracture	No	659 (80.0)	22.0	0.96
	Yes	165 (20.0)	21.8	
Prior non-vertebral osteoporotic fracture	No	782 (94.9)	21.4	0.07
	Yes	42 (5.1)	33.3	
Morphometric vertebral fracture	No	680 (84.4)	21.0	0.002
	Mild	76 (9.4)	18.4	
	Mod/Severe	50 (6.2)	42.0	
Glucocorticoid treatment	No	773 (93.8)	21.5	0.19
	Yes	51 (6.2)	29.4	
Other drugs that decrease bone mass	No	756 (91.8)	22.0	0.99
	Yes	68 (8.3)	22.1	
Smoking	No	788 (95.6)	22.1	0.71
	Yes	36 (4.4)	19.4	
Dietary calcium intake	≥500mg/day	761 (92.4)	22.3	0.37
	<500mg/day	63 (7.7)	17.5	
Other secondary causes of osteoporosis	No	726 (88.1)	21.9	0.90
	Yes	98 (11.9)	22.5	
FRAX 10-years risk hipfracture^c^	≤1	545 (66.1)	19.3	0.02
	1–3	170 (20.6)	28.8	
	>3	109 (13.2)	24.8	
TOTAL [unweighted]		824(100.0)	22.0	

*BMI, body mass index; BMD, bone mass density*.

^*a*^ *n = 824; missing data: studies (123), vertebral fracture (18), BMI (1), BMD (1)*.

^*b*^ *χ2 test*.

^*c*^ *FRAX scores were calculated using the BMD results*.

*Treatment prevalence weighted to represent the age-structure of women of 50 and over in*

*Valencia was 20.9% (95%CI: 17.6–24.4)*.

In the multivariable analysis (Table 2), the factors independently associated with the prescription of antiosteoporotic drugs were: early menopause (2.6 times greater odds of having an antiosteoporotic drug prescription), morphometric moderate or severe vertebral fractures (2.7 times greater odds), and densitometric osteoporosis (1.5 greater odds). Women aged 65–69 years old were also more likely to have a prescription than women in the lowest age group (reference group). Obesity worked as a factor reducing the likelihood of being treated.

**Table 2:** Factors associated with antiosteoporotic treatment in postmenopausal women. **Multivariable logistic regression analysis.** ^a^ ^,^ ^b^

	OR	95%CI		p-value
Age 65–69 years (ref. 50–55 years)	1.60	1.06	2.42	0.02
BMI ≥30 (ref. 20–25)	0.45	0.30	0.67	<0.001
Menopause age≤40 (ref 40 and over)	2.63	1.55	4.50	<0.001
Vertebral fract. mod/severe (ref. no fracture)	2.72	1.47	5.04	0.001
Densitometric osteoporosis (ref. normal T-Score)	1.51	1.05	2.19	0.03

^*a*^ *OR*, *odds ratio; 95%CI*, *95% Confidence Interval*.

^*b*^ *n = 804; Pseudo r* ^*2*^ *= 0*.*06; p<0*.*0001; C-Statistic*: *0*.*66; p(X* ^*2*^ *) Hosmer–Lemeshow = 0*.*598*.

Regarding the potential impact on the population of applying the criteria for treatment of the CPGs analyzed (Table 3), between 8.7% (Osteoporosis Canada guideline) and 36.6% (National Osteoporosis Foundation guideline) of women would be recommended for treatment according to the international CPGs, while the percentage of women of 50 years old and over that would be treated according to the Spanish CPGs would range between 17.7% (Spanish Society for Family and Community Medicine guideline) and 44.3% (Spanish Rheumatology Society guideline).

**Table 3:** Impact on the population and inappropriateness according to osteoporosis guidelines’ recommendations for treatment.^a^

		Women recommended for treatment		Inappropriateness^b^	
		%	95%CI	% Overuse	% Underuse
International CPGs	Osteop. Canada	8.7	5.8–11.6	72.6	6.6
	NOGG (UK)	10.8	7.9–13.8	77.8	7.9
	NICE (UK)	13.9	10.7–17.2	73.4	10.7
	NOF (US)	36.6	33.1–40.1	56.4	34.6
Spanish CPGs	semFYC	17.7	14.4–21.1	64.9	14.3
	SNS	19.4	15.9–22.9	55.5	17.3
	SEIOMM	20.6	17.1–24.1	66.0	17.2
	SEMERGEN	24.1	20.4–27.8	57.9	19.4
	SECOT	29.9	26.5–33.2	50.6	28.1
	SER	44.3	40.7–47.8	41.5	41.3

^*a*^ *CPGs*, *Clinical Practice Guidelines; CANADA*, *Osteoporosis Canada; NICE*, *National Institute for Health and Care Excellence; NOGG*, *National Osteoporosis Guideline Group; NOF*, *National Osteoporosis Foundation; semFYC*, *Spanish Society for Family and Community Medicine; SNS*, *Spanish National Health System; SEIOMM*, *Spanish Society for Bone Research and Mineral Metabolism; SEMERGEN*, *Spanish General Medical Society; SECOT*, *Spanish Society for Orthopaedic Surgery and Traumatology; SER*, *Spanish Rheumatology Society*.

^*b*^ *The percentage of overuse is based on the population treated (n = 181)*, *and the percentage of underuse is based on the population of untreated women (n = 643)*.

**Fig 2:**
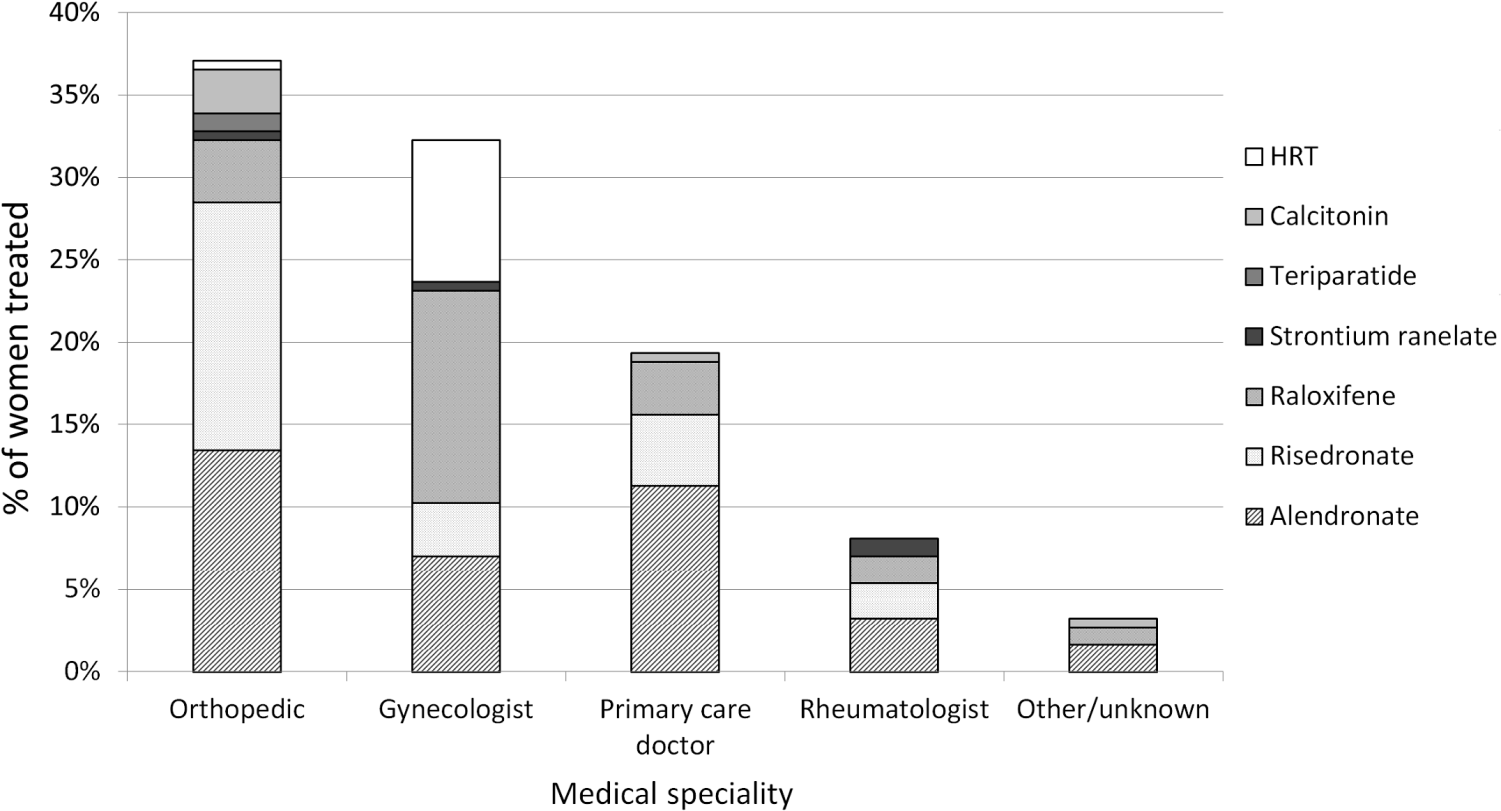
Antiosteoporotic treatments according to the medical specialty responsible for the initial prescription. Abbreviations: HRT, Hormone Replacement Therapy.

Regarding inappropriateness of treatments (Table 3), between 56.4% and 77.8% of women under treatment did not meet any criteria to be treated according to the international CPGs; these figures ranged from 41.5% to 66.0% when we applied the Spanish CPGs criteria. According to all guidelines, the overuse of antiosteoporotic treatments in postmenopausal women of 50 years old and over was 45.3% (61.3 and 46.4% according to the international and Spanish CPGs, respectively, Fig 2). Concerning the underuse, between 6.6% and 34.6% of untreated women should have received treatment according to the international CPGs, whereas these figures were between 14.3% and 41.3% when the Spanish CPGs were applied. According to all guidelines, the underuse of antiosteoporotic treatments in postmenopausal women of 50 years old and over was 0.9% (3.4 and 2.0% according to the international and Spanish CPGs, respectively, Fig 2).

## Discussion

Our study shows that the prevalence of antiosteoporotic treatment in postmenopausal women ≥ 50 in Valencia was 20.9% and the type of antiosteoporotic drugs prescribed varied greatly depending on the medical specialty responsible of the initial prescription. The impact on the population and the proportion of treatments considered inappropriate when applying the most influential osteoporosis guidelines, varied strikingly, with the percentage of women 50 and over who should be treated ranging from less than 9% to over 44%. A large proportion of inappropriate treatments was found when applying these guidelines to the Spanish population, combining a high overuse (which ranged between 42 and 78%) and, to a lesser extent, underuse (ranging between 7 and 41%).

In the city of Valencia, one out of five women aged 50 and older were treated with antiosteoporotic drugs. This treatment rate, lower than that reported for the Valencia region in 2010[15], may be related to the healthier characteristics of a real population sample (enrolled from a population registry, not from medical practices), increasing treatment rates during the time period between the two studies (the dispensing of antiosteoporotics in Spain rose by nearly 50% between 2006 and 2008[14]) or real differences in treatment rates between the city of Valencia and the rest of the Valencia region. Nevertheless, our results show high treatment rates in women with a low to moderate risk of fracture (19% and 29% in women with≤1% and 1–3% 10-year risk of hip fracture assessed by FRAX, respectively), and a huge proportion of unnecessary treatments (between 42% and 78%) according to the criteria of the most influential CPGs. These figures suggest a striking amount of osteoporosis treatment overuse and an interesting opportunity to reduce costs (including those related to adverse events from unnecessary treatments) without compromising-and probably improving- patients’ health.

Regarding treatment underuse (7–41%, depending on the guideline used), although lower than the treatment overuse observed in relative terms, it is based on a larger population (the untreated women, 79%), also becoming a major issue in osteoporosis management. Some of the figures described in the bivariate analysis were highly suggestive of underuse. Although some acknowledged risk factors are associated with an increased likelihood of being treated, other recognized risk factors did not show such an association, or the proportion of women treated is too sparse: only 33% of women with prior non-vertebral fractures and 42% of women with moderate-severe vertebral fractures were receiving osteoporosis treatment. Even some risk factors such as age or FRAX 10-year risk of hip fracture showed paradoxical behaviour, with a greater likelihood of treatment at intermediate values but no association with the highest-risk scores. These figures confirm the existence of a relevant “osteoporosis care gap” in the Spanish setting, something which has already been revealed in other countries[37,38],and has remained unchanged over time [39,40].

Our study also shows the dramatic impact on the population treatment rates of applying the diverse CPGs recommendations, varying the percentage of women who should be treated according to different guidelines from less than 9% to over 44%. In real terms, from the approximately eight million women of 50 years old and over in Spain, the number eligible for treatment would range from 0.7 to 3.8 million, depending on the guideline used. These results are consistent with those reported by Bolland and Gray, showing treatment recommendations for 21% and 48% of women after applying the NOGG and NOF guidelines criteria, respectively, in a cohort of older women (mean age74 years) participating in a clinical trial in New Zealand [10] (which would be 11% and 37% for such CPGs in our study population, which is 10 years younger).

Treatment decisions and the choice of a particular drug could be influenced by patient characteristics, physician and organizational factors, pharmaceutical promotion and healthcare system characteristics[41]. One interesting result of our study-limited by the small number of cases for analysis-is that the selection of the specific antiosteoporotic agent seems to be more dependent on the specialty of the physician starting treatment than on patient characteristics. Several studies have found that the pharmacological management of several conditions varies greatly by physician speciality [42]; however, the quality and relevance of those studies has been criticized. Moreover, little is known regarding this variability in the pharmacological management of osteoporosis. In our study, the prescribing patterns of gynaecologists were particularly remarkable, treating only one third of patients with bisphosphonates and almost two thirds with raloxifene (40%) or hormone replacement therapy (25%)–five years after the publication of the Women's Health Initiative trial results [43]. These results could be explained, at least in part, because these specialists often treat younger postmenopausal women. In this sense, it is likely that such prescription patterns could be a significant source of overuse, given the low risk of osteoporotic fracture in young women, although it could also be a strictly local finding. Furthermore, we found that orthopaedic surgeons were responsible for most of the initial antiosteoporotic prescriptions. This might be explained by the relative lack of rheumatologists within the Spanish National Health System, and the consequent tendency of primary care physicians to refer these patients to the orthopaedic surgeons.

### Limitations

Our study has some potential limitations. First, we “applied” several U.S., U.K., and Canadian guidelines to a Spanish population that may have different characteristics (e.g. prevalence of risk factors, incidence of fracture, strength of the associations between risk factors and the incidence of fracture, etc.) than the populations where the decision rules have been developed. Second, although the dropout rate in the FRAVO study is similar to other population studies, it was higher in the oldest group (with higher expected morbidity) but also in younger working women, who were presumably healthier [18]. Weighting for age should have partially reduced this limitation, but it is difficult to assess the effect and scope of any possible biases linked to missed cases in recruitment. Third, the selection of particular guidelines from among the myriad of existing documents (the International Osteoporosis Foundation website includes links to more than 50 guidelines; see: http://www.iofbonehealth.org/guideline-references) always has a subjective component, although we consider that those selected are among the most representative, widely used, and influential in Spain, Europe, the United States and Canada. Furthermore, these guidelines were also rated by physicians of different specialities as the most influential in their clinical practice. Fourth, some of the risk factors considered in the CPGs evaluated were not available or had different definitions in the FRAVO data or some CPGs include vague criteria that are impossible to operationalize unambiguously. We operationalized these criteria, as indicated in Table A in S1 File, using "reasonable" interpretations, but other possible interpretations may have led to different estimates of population impact and inappropriateness.

## Conclusions and Implications

The pharmacological management of osteoporosis in women of 50 and over combines an important overuse (mainly in young women with low risk of fracture) with an important underuse (in women who are older, at high risk or with previous osteoporotic fractures), although the level of inappropriateness varies dramatically depending on the CPGs used. In a recent paper [25] we described the high variability among CPGs in recommending antiosteoporotic treatment, concluding that such variability limits the effectiveness of those recommendations and, given the heterogeneity of the criteria used, it should come as no surprise that doctors and health care providers could become confused to the point of inaction or misguided action. In the present study, we quantified the impact of this variability on the number of women who should be treated and on the inappropriateness of the prescribed treatments, and the overwhelming results should not be overlooked by public health care policies in Spain (and probably in other countries in similar situations) because osteoporosis is a frequent condition and even small variations in treatment indication can account for large differences in women treated and resources consumed.

Targeting high-risk populations is a strategic element for developing cost-effective policies in the prevention of osteoporotic fractures. Predictive modelling of fracture risk factors seems to be the main instrument for stratifying the population into risk groups to which practical policies should be applied, and CPGs should help integrate this information to identify people who are more likely to benefit from treatment. The results of our study suggest that the current CPGs, although based on the same evidence, seem to interpret it differently and do not meet these clinical and policy needs sufficiently. The development of more accurate predictive tools (especially for the intermediate risks) could possibly contribute to the convergence of these interpretations, to a consensus on more homogeneous guidelines and, eventually, to the reduction of osteoporotic fractures. However, at present, it seems urgent to develop policies to reduce treatment overuse (at least in those cases where there is wide agreement) while reducing underuse should also not be neglected, especially in women in secondary prevention for whom, beyond their scores of predictive risk tools, a high risk of osteoporotic fracture has already been shown.

## Supporting Information

S1 File Online Supporting Information. Covariate definitions**(Appendix A)**. Framework for assessing overuse and underuse**(Figure A).** Criteria for antiosteoporotic treatment according to the guidelines selected**(Table A).**
(DOC)

S1 Dataset (CSV)
