# Supplementary material for: Overuse and Underuse of Antiosteoporotic Treatments According to Highly Influential Osteoporosis Guidelines: A Population-Based Cross-Sectional Study in Spain
Source: PLoS One. 2015 Aug 28;10(8):e0135475. doi: 10.1371/journal.pone.0135475 (PMC4552751; doi:10.1371/journal.pone.0135475)
Supplement: S1 File — Covariate definitions(Appendix A). Framework for assessing overuse and underuse(Figure A). Criteria for antiosteoporotic treatment according to the guidelines selected(Table A). (DOC) [file pone.0135475.s001.doc]

**S1 File. Online Supporting Information**

**Appendix A.** Covariate definitions

Treatment with glucocorticoids: use of oral glucocorticoid for at least 3 months in the previous year.

Other drugs that decrease bone mass: at least one prescription of lithium, anticonvulsants, high dose thyroxin or immunosuppressive treatment in the previous year.

Smoking: defined as a smoking history of ≥20 cigarettes per day

Other secondary causes of osteoporosis: gastrectomy, bowel resection, inflammatory bowel disease, thyroidectomy, diabetes mellitus, chronic liver disease, chronic obstructive pulmonary disease, rheumatoid arthritis, transplantation, chronic kidney failure.

| **Figure A. Framework for assessing overuse and underuse** | | | | |
| --- | --- | --- | --- | --- |
|  | | | | |
|  |  | **Appropriateness criteria** | | |
|  |  | **Service**  **recommended** |  | **Service not**  **Recommended** |
| **Healthcare service**  **Provided** | **Yes** | *Appropriate*  *utilization* | **Gray area** | ***Overuse*** |
|  | **No** | ***Underuse*** |  | *Appropriate*  *non-utilization* |
| *The gray area may be due to lack of evidence, lack of specificity of the appropriateness criteria, or lack of information on the status of the particular patient.* | | | | |

| **Table A. Criteria for antiosteoporotic treatment according to the guidelines selected** | |
| --- | --- |
| **Guideline Criteria** | **Operativization** |
| **NOF 2010**^1^ | |
| Women aged 50 and over with a hip or vertebral (clinical or morphometric) fracture | ✓ |
| Women aged 50 and over with T-score ≤ -2.5 at the femoral neck or spine after appropriate evaluation to exclude secondary causes | ✓ |
| Women aged 50 and over with low bone mass (T-score between -1.0 and -2.5 at the femoral neck or spine) and a 10-year probability of hip fracture ≥ 3% or a 10-year probability of a major osteoporosis-related fracture ≥ 20% based on the US-adapted WHO algorithm | ✓ |
|  | |
| **Canada 2010**^2^ | |
| Women at **high absolute risk** (FRAX 10-year probability of a major fracture ≥ 20%) | ✓ |
| Women over 50 years old who have had a hip or vertebral fragility fracture | ✓ |
| Women over 50 years old who have had more than one fragility fracture | We do not have information available regarding multiple fractures at the same location. We only considered more than one fragility fracture in different locations. |
| **Women with moderate risk** (FRAX 10-year probability of a major fracture 10%–20%), should be considered for pharmacologic treatment. Additional risk factors should be used to guide pharmacologic therapy:  •Additional vertebral fracture(s) (by vertebral fracture assessment or lateral spine radiography)  • Previous wrist fracture in individuals aged > 65 years old and those with a T-score ≤ –2.5  • Lumbar spine T-score << femoral neck T-score  • Rapid bone loss  • Women undergoing aromatase inhibitor therapy for breast cancer  • Long-term or repeated use of systemic glucocorticoids (oral or parenteral) not meeting conventional criteria for recent prolonged use  • Recurrent falls (≥ 2 in the past 12 months)  • Other disorders strongly associated with osteoporosis, rapid bone loss or fractures | We have considered these women with ambiguous criterion (gray area, **Figure A in S1 File**) |
|  | |
| **NICE 2011**^3^ | |
| Women aged 75 and over who have had a fragility fracture | ✓ |
| Women aged 75 and over with ≥ 2 risk factors for fracture^a^ or with ≥ 2 risk factors for low BMD^b^ | ✓ |
| Women aged 75 and over with ≥1 risk factor for fracture^a^ and who have a T-score ≤ -2.5 | ✓ |
| Women aged <75 years old who have had a fragility fracture and a T-score ≤ -2.5 | ✓ |
| Women aged 70-75 years old who have a T-score ≤ –2.5 and with ≥1 risk factor for fracture^a^ or with ≥1 risk factor for low BMD^b^ | ✓ |
| Women aged 65-69 years old who have a T-score ≤ –2.5 and with ≥1 risk factor for fracture | ✓ |
| Women aged <65 years old who have a T-score ≤ –2.5 and with ≥1 risk factor for fracture^a^ and ≥1 risk factor for low BMD^b^. | ✓ |
|  | |
| **S1Table. Continued** | |
| **Guideline Criteria** | **Operativization** |
| **NOGG 2010**^4^ | |
| Based on intervention thresholds using age and FRAX 10-year probability of a major or hip fracture. | We used the FRAX calculation tool for Spain and the NOGG guideline thresholds (there are no specific thresholds for Spain). |
|  |  |
| **SEMERGEN 2006**^5^ | |
| Women with multiple vertebral fractures or a hip fracture | We do not have information available about “multiple vertebral fracture”. We have only considered the presence of a hip fracture. |
| Women with vertebral fracture and aged 65 years old and over | ✓ |
| Women with vertebral fracture and aged < 65 years old and a T-score ≤ –1.5 | ✓ |
| Women with non vertebral fracture and a T-score ≤ –1.5 | ✓ |
| Women with CRFs^c^ and a T-score ≤ –2.5 | We have considered at least one CRFs^c^ and T-score ≤ –2.5 |
| Women of 50 and over with glucocorticoid treatment (At least three months cumulative therapy in the previous year at a prednisone-equivalent dose ≥ 7.5 mg daily) | For glucocorticoid treatment we have considered at least three months cumulative therapy in the previous year at a prednisone-equivalent dose ≥ 5 mg daily |
|  | |
| **semFYC**^6^ | |
| Women who have had a fragility fracture or used systemic glucocorticoid (At least three months cumulative therapy in the previous year at a prednisone-equivalent dose ≥ 5mg daily) | ✓ |
| Women aged 65 and over with a T-score ≤ –2.5 (taking into consideration low T-score values and increased age/older ages and also the presence of low body mass index -defined as less than 19 kg/m2-, history of hip fracture in mother and high fall risk) | We have considered women aged 65 and over with T-score ≤ –2.5 with or without risk factors as appropriately treated |
| Women aged 65 y. and older with a T-score between -1.0 and -2.5: In general treatment is not recommended but consider pharmacologic therapy if T-score < –2.0 values and presence of other CRFs^d^ | We have considered these women with ambiguous criterion (gray area, **Figure A in S1 File**) |
| Women aged ≤65 with a T-score ≤ –2.5: Consider pharmacologic therapy at low T-score values and also taking into account the presence of CRFs^d^ | We have considered these women with ambiguous criterion (gray area, **Figure A in S1 File**) |
|  | |

| **S1Table. Continued** | |
| --- | --- |
| **Guideline Criteria** | **Operativization** |
| **SEIOMM 2008**^7^ | |
| Women with a T-score ≤ –2.5 | ✓ |
| Women who have had a vertebral fracture | ✓ |
| Women who have had a non-vertebral fracture (hip or wrist) and T-score ≤ –2.5 | ✓ |
| Women aged 65 and over who use of systemic glucocorticoids (At least three months cumulative therapy in the previous year at a prednisone-equivalent dose ≥ 7.5 mg daily) | For glucocorticoid treatment we have considered at least three months cumulative therapy in the previous year at a prednisone-equivalent dose ≥ 5 mg daily |
| Women aged ≤65 who use systemic glucocorticoids and with a T-score ≤ –1.5 | ✓ |
|  | |
| **SECOT 2010**^8^ | |
| Women who have had a fragility fracture | ✓ |
| Women without previous fragility fracture: high risk (OST score^e^ < -2) or moderate risk (OST score ≥-2 ≤ -1) and a T-score ≤ -2.5 | ✓ |
| Consider pharmacological treatment for women with a T-score ≤ –2.5 not included in previous criteria | We have considered these women with ambiguous criterion (gray area, **Figure A in S1 File**) |
|  | |
| **SER 2011**^9^ | |
| Women who have had a fragility fracture | ✓ |
| Women aged 65 and over who use systemic glucocorticoids (At least three months cumulative therapy in the previous year at a prednisone-equivalent dose ≥ 5 mg daily) | ✓ |
| Women aged < 65 who use systemic glucocorticoids and has a T-score ≤ -1.5 | ✓ |
| Women with a T-score ≤ -2.5 taking into consideration their CRFs^f^ | We have considered women with a T-score ≤ -2.5 and with ≥1 CRF^f^ as appropriately treated |
| Consider pharmacological treatment for women with premature menopause (<45 years old), taking into account T-score values and other CFRs^f^ | We have considered these women with ambiguous criterion (gray area, **Figure A in S1 File**) |
| Consider pharmacological treatment in women with a T-score between -1.0 and -2.5 only in specific cases (e.g. young women with a T-score near to ≤ –2.5 with other CRFs^f^). | We have considered these women with ambiguous criterion (gray area, **Figure A in S1 File**) |
|  | |
| **SNS** ^10^ | |
| Women who have had a fragility fracture and a T-score ≤ –2.5 | ✓ |
| Women without previous fragility fracture who have ≥ 2 CRFs^g^ and with T-score ≤ –2.5 | ✓ |
| Consider pharmacological treatment in women without previous fragility fracture who have ≥ 2 CRFs^g^ and a T-score between -1.5 and -2.49 | We have considered these women with ambiguous criterion (gray area, **Figure A in S1 File**) |
| Abbreviations: CRFs, clinical risk factors.  ^a^NICE risk factors for fracture: parental history of hip fracture, alcohol intake ≥4 units per day and rheumatoid arthritis.  ^b^NICE risk factors for low BMD: low body mass index (defined as less than 22 kg/m2), untreated premature menopause, and secondary causes of osteoporosis (gastrectomy, bowel resection, inflammatory bowel disease, thyroidectomy, diabetes mellitus, chronic liver disease, rheumatoid arthritis, transplantation).  ^c^SEMERGEN CRFs: 65 years and older, low body mass index (defined as less than 20 kg/m2), untreated premature menopause (<45 years old), history of hip fracture in mother, secondary causes of osteoporosis (rheumatoid arthritis, untreated long-standing hyperthyroidism, chronic malnutrition or malabsorption, coeliac disease and others).  ^d^semFYC CRFs: age, low body mass index (defined as less than 19 kg/m2), parental history of hip fracture and high risk of falls.  ^e^OST (Osteoporosis Self-Assessment Tool), OST score was calculated as 0.2 × (weight in kg - age in years).  ^f^SER CRFs: age 65 and older, parental history of hip fracture, low body mass index (defined as less than 20kg/m2), personal history of fracture or risk of falls (≥ 2 in the past 12 months).  ^g^SNS CRFs: age 65 and older, parental history of hip fracture, low body mass index (defined as less than 20kg/m2 ), glucocorticoid treatment (at least three months cumulative therapy in the previous year at a prednisone-equivalent dose ≥ 5 mg daily), premature menopause, falls in the past 12 months, hyperparathyroidism, eating disorders (anorexia, bulimia) and inflammatory bowel disease. | |
